# Exogenous progesterone supplementation: a strategy to enhance conceptus development in sheep and pigs?

**DOI:** 10.1530/RAF-24-0092

**Published:** 2025-01-11

**Authors:** Maria F Tyree, Claire Stenhouse

**Affiliations:** Department of Animal Science, Pennsylvania State University, University Park, Pennsylvania, USA

**Keywords:** pregnancy, uterus, fetus, embryo, endocrinology of reproduction

## Abstract

**Abstract:**

The inability of animals to get pregnant, pregnancy loss and weak or stillborn offspring are significant economic burdens to livestock producers worldwide. Progesterone, the hormone of pregnancy, has a crucial role in the establishment of pregnancy, and it has been suggested that progesterone supplementation may be a promising strategy to improve pregnancy outcomes and conceptus development. This review article describes the existing literature on progesterone supplementation in sheep and pigs in relation to pregnancy outcomes and conceptus development.

**Lay Summary:**

Pregnancy loss and weak offspring are significant problems both in humans and in agriculturally relevant species. Progesterone (the hormone of pregnancy) is important for establishing pregnancy and regulating fetal and placental growth, and it is speculated that progesterone supplementation could be a promising method to improve pregnancy outcomes and both fetal and placental growth. This review article describes the existing literature on progesterone supplementation in sheep and pigs in relation to pregnancy outcomes and fetal and placental development.

## Introduction

The establishment and maintenance of pregnancy requires precise cell- and time-specific alterations in the expression of many genes by both the endometrium and conceptus (embryo/fetus and associated extraembryonic membranes) (Almeida & Dias 2022, Stenhouse *et al.* 2022). In livestock species, this intricate process frequently fails and the inability of animals to get pregnant, pregnancy loss and weak or stillborn offspring are some of the largest economic burdens to livestock producers worldwide. It is estimated that 20–30% of ovine pregnancies fail, with two-thirds of these losses occurring during the peri-implantation period of pregnancy (Nancarrow 1994, Diskin and Morris 2008, O’Connell *et al.* 2016). While pregnancy rates are generally greater in pigs than in many other livestock species, spontaneous embryonic/fetal loss remains a significant concern worldwide. In fact, it is estimated that approximately 30% of genetically normal pig conceptuses are lost (Pope *et al.* 1990, Tayade *et al.* 2006, Bidarimath & Tayade 2017, Zang *et al.* 2021). Similar to sheep, much of this loss occurs during the peri-implantation period (days 12–30 of pregnancy) (Stroband & Van der Lende 1990, Geisert & Schmitt 2002, Ross *et al.* 2009), which is followed by a subsequent period of fetal loss in mid-late gestation (days 50–90) (Vonnahme *et al.* 2002, Vallet *et al.* 2011). While the exact incidence of pregnancy loss or embryonic/fetal mortality varies across these species, there is a consistent observation of high embryonic mortality in early pregnancy, suggesting that similar mechanisms regulate this phenomenon.

As discussed below, the production of sufficient amounts of the steroid hormone progesterone (P4) by the corpus luteum (CL) is required for the establishment of pregnancy in livestock species. Given the critical function of P4 in early pregnancy, a period associated with extensive pregnancy loss, several studies have investigated the potential of P4 supplementation as a strategy to enhance pregnancy outcomes and conceptus development. To date, there are conflicting reports surrounding the impact on pregnancy outcomes, conceptus development and the optimal timing, dosage and routes of administration. This review will summarize the current understanding of the potential of P4 supplementation as a strategy to enhance pregnancy outcomes and conceptus development in sheep and pigs.

## Progesterone as a regulator of uterine function

Sheep and pigs have a 17- and 21-day estrous cycle, respectively (Bazer *et al.* 1998). The estrous cycle in these species is composed of a follicular phase (characterized as a period of estrogen dominance), which includes the proestrus and estrus stages, and a luteal phase (characterized as a period of P4 dominance), which includes the metestrus and diestrus stages. Following ovulation, the newly formed corpus luteum (CL) produces P4. In a non-pregnant animal, P4 production and secretion will decline during proestrus due to regression of the CL (luteolysis), resulting in a switch from P4 to estrogen dominance.

To establish and maintain pregnancy, the conceptus must signal the mother to maintain the CL prior to the initiation of luteolysis. This early conceptus signaling is known as the maternal recognition of pregnancy. In ruminants, the conceptus releases interferon tau (IFNt), which blocks the luteolytic action of prostaglandin F2 alpha (PGF2a) (Bazer *et al.* 1991). In pigs, the maternal recognition of pregnancy requires sequential release of estrogen, interleukin-1B2, prostaglandin E2 and interferon-gamma (Geisert *et al.* 2024). Once maternal recognition of pregnancy occurs and luteolysis is prevented, P4 production by the CL is maintained.

Pigs exhibit diffuse epitheliochorial placentation in which the trophectoderm cells of the conceptus attach to the uterine luminal epithelium and both the chorionic epithelium and uterine luminal epithelium remain intact for the duration of gestation (Johnson *et al.* 2021). The CL act as the primary source of progesterone for the entire pregnancy in pigs (Ziecik *et al.* 2018). In contrast, sheep exhibit more invasive synepitheliochorial placentation (Seo *et al.* 2024) and the placenta takes over from the CL as the primary producer of progesterone between days 50 and 70 of gestation (Ricketts & Flint 1980). However, despite these differences in placentation strategy, P4 is essential for the establishment and maintenance of pregnancy in both species.

Progesterone is a cholesterol-derived steroid hormone composed of 21 carbon atoms (Taraborrelli 2015). Progesterone has several effects on the uterus, including the suppression of the myometrium into a quiescent state and the promotion of histotrophic secretions (nutrient-rich secretions from the uterine epithelia contain hexose sugars, growth factors, water, vitamins, amino acids, ions, hormones and minerals), all of which are necessary to support the growth and development of the conceptus(es) prior to implantation and the establishment of a fully functional placenta (Bazer *et al.* 2008). The actions of P4 on the female reproductive tract are mediated through binding of P4 to its receptor (PGR) in the uterus. Once bound, the P4–PGR complex undergoes dimerization and translocation into the nucleus where it can bind to DNA to regulate gene expression, leading to downstream effects on cellular function (Wetendorf & DeMayo 2014).

Prolonged P4 exposure during the pre-implantation period results in downregulation of PGR in both luminal (LE) and glandular epithelium (GE) of the endometrium (Spencer & Bazer 1995). The downregulation of PGR expression in epithelia is required across eutherian mammals for implantation and results in alterations in gene expression by the uterine epithelia that are responsible for changes in histotrophic secretions, which support the conceptus prior to implantation (Bazer 1975). In contrast, PGR expression is maintained in both the endometrial stroma and the myometrium throughout gestation in sheep and pigs (Spencer *et al.* 2004).

## Review of studies investigating exogenous progesterone supplementation in pregnancy

Given the critical function of P4 in early pregnancy, a period associated with extensive pregnancy loss, several studies have investigated the potential of P4 supplementation as a strategy to enhance pregnancy outcomes and conceptus development in sheep and pigs.

### Sheep

Studies undertaken to determine the impact of exogenous P4 supplementation on conceptus development in sheep have yielded highly variable results, with studies suggesting that P4 supplementation has no effect on, has negative effects on, or enhances pregnancy outcomes. These contradictory findings appear to be largely influenced by timing, frequency and dosage of supplementation.

Several studies have suggested that exogenous P4 supplementation in early pregnancy in sheep accelerates conceptus development. Ewes supplemented with daily exogenous P4 by intramuscular injection (i.m., 25 mg on days 1.5–9) had a greater blastocyst diameter on day 9 of gestation compared to controls (Satterfield *et al.* 2006). Similarly, on day 12 of gestation, ewes supplemented with P4 had conceptuses with a filamentous morphology, whereas conceptuses from control ewes had a spherical morphology (Satterfield *et al.* 2006). While exogenous P4 treatment appears to accelerate pre-implantation conceptus development, the impact of this enhanced development in early pregnancy on conceptus growth and development in late pregnancy remains poorly understood.

In sheep, histotrophic secretions from the endometrium are an essential nutrient source for the developing conceptus, particularly before the establishment of the placenta. These secretions contain various nutrients, growth factors and enzymes vital to early conceptus development and maintenance of pregnancy (Gray *et al.* 2002). Histotrophic secretions are largely regulated by actions of P4 on the uterine endometrium (Spencer & Bazer 2002). Similarly, it has been suggested that exogenous P4 alters the expression of genes in the uterine LE and GE, resulting in alterations in the composition of histotrophic secretions released into the uterine lumen (Satterfield *et al.* 2006).

Exposure of the uterus to P4 during early pregnancy downregulates the expression of the PGR in uterine epithelia, leading to changes in uterine gene expression (Bazer 1975). This is supported by the finding that certain P4-induced genes, such as galectin 15 (*LGAL15*), expressed in the endometrium and their products secreted into the uterine lumen are upregulated in sheep supplemented with exogenous P4. LGAL15 is hypothesized to regulate trophectoderm proliferation and adhesion during the implantation period (Gray *et al.* 2004, Lewis *et al.* 2007). Previous research has demonstrated that under normal physiological conditions, LGAL15 does not undergo upregulation in the endometrium until days 10–12 of pregnancy, coinciding with the period of PGR downregulation in the epithelia (Gray *et al.* 2004). Exogenous P4 supplemented on days 1.5 to 9 resulted in an increase in both expression of *LGAL15* mRNA in the uterine LE and GE and a greater abundance of LGAL15 protein in uterine flushings on day 9 of pregnancy (Satterfield *et al.* 2006). This suggests that P4 supplementation in early pregnancy induces alterations in the expression of mRNAs with known roles in the regulation of conceptus growth and development in the endometrium.

Similarly, when exogenous P4 is administered during early gestation, the downregulation of the PGR in the epithelia is also advanced, leading to an asynchronous uterine environment (Bazer 1975). Synchrony between the early blastocyst and the uterine endometrium is critical for implantation (Wilmut & Sales 1981). Thus, it is possible that this accelerated conceptus development and advanced downregulation of the PGR in the uterus may result in asynchrony between the conceptus and the uterus. In fact, some studies have suggested that P4 supplementation in early pregnancy may result in increased pregnancy loss. Supplementation of ewes with P4 using controlled internal drug release (CIDR) starting on the day of insemination (days 0–17) or three days post-insemination (days 3–17) resulted in increased pregnancy loss on day 54 in ewes given P4 starting on day 0 compared to controls and to ewes supplemented with P4 starting on day 3 (Rickard *et al.* 2017). Furthermore, sheep supplemented withP4 on days 1.5–8 (i.m., 25 mg) had lower pregnancy rates on day 35 of gestation compared to control sheep that were not exposed to exogenous P4 (Halloran *et al.* 2021). This suggests that P4 supplementation, particularly immediately after breeding, may have negative consequences for pregnancy outcomes. In contrast, Satterfield and coworkers demonstrated an increase in both IFNt abundance in uterine flushings and greater expression of mRNAs encoding interferon-stimulated genes in the endometrium on day 12 from ewes treated with P4 compared to endometria from control ewes (Satterfield *et al.* 2006). Under normal physiological conditions, IFNt secretion increases from day 12, with peak secretion between days 15 and 17 of pregnancy (Forde & Lonergan 2017). It is possible that this advanced increase in IFNt secretion by the conceptus is an attempt to maintain synchrony between the conceptus and the uterus, thereby allowing the establishment and maintenance of pregnancy (Satterfield *et al.* 2006). However, there are several unexplored factors including alterations in utero-conceptus signaling that could affect the synchrony between the conceptus and the uterus in response to exogenous P4 that require further investigation.

The rapidly developing conceptus requires extensive proliferation, remodeling and migration, which demand significant energy expenditure. Glucose and fructose are extensively utilized by the conceptus to support these processes (Moses *et al.* 2022). As noted above, P4 supplementation in early gestation enhanced blastocyst development and elongation (Satterfield *et al.* 2006). Therefore, it could be hypothesized that P4 modulates the transport of glucose and fructose into the uterine lumen as a way of supporting this accelerated rate of development. In fact, supplementation with exogenous P4 in early pregnancy results in alterations in the expression of mRNAs encoding glucose transporters in the endometrium. Supplementation with P4 on days 1.5–8 (i.m., 25 mg) increased the expression of solute carrier family 2 member 1 (*SLC2A1*) mRNA (also known as GLUT1) on both days 9 and 12 of gestation (Satterfield *et al.* 2010). Similarly, the expression of *SLC2A1* and *SLC2A5* (fructose transporter) mRNAs was greater in the endometrium on day 125 of pregnancy following P4 administration on days 1.5–8 (i.m., 25 mg) (Halloran *et al.* 2021). These changes were not detected in early pregnancy, with no significant difference in the expression of mRNAs encoding glucose transporters in endometria on days 9 or 12 of pregnancy following supplementation of P4 (i.m., 25 mg) on days 1.5–8 (Hoskins *et al.* 2021). Although a tendency for increased glucose in uterine flushings has been reported, no significant increase in glucose has been found in histotrophic secretions, allantoic fluids or amniotic fluids of P4-treated ewes (Satterfield *et al.* 2006, Halloran *et al.* 2021, Hoskins *et al.* 2021). It is possible that the lack of increased glucose results from greater glucose utilization by the conceptus. However, it is unclear whether a change in mRNA expression of glucose transporters leads to a change in glucose availability and/or utilization during pregnancy and further investigation is required.

Similarly, the utilization of ovariectomized cyclic ewes treated with P4 (i.m., 50 mg daily) on days 5–16 increased the expression of mRNAs encoding the glucose transporters *SLC2A4* and *SLC5A1* in the uterine LE and superficial glandular epithelium (sGE) when compared to control groups given P4 in conjunction with a PGR antagonist, suggesting a regulatory role of P4 through binding to PGR on the expression of glucose transporters in the ovine uterus (Gao *et al.* 2009). In contrast, ewes given P4 (i.m., 50 mg daily) on days 5–16 in combination with intrauterine infusions of IFNt increased the expression of mRNAs encoding the glucose transporters *SLC2A1* and *SLC5A11* in the uterine LE and sGE when compared with endometria from ewes treated with P4 alone and ewes treated with P4 plus a PGR antagonist (Gao *et al.* 2009). Collectively, this study suggests that while the expression of mRNAs encoding the glucose transporters *SLC2A4* and *SLC5A1* is regulated by P4 binding to PGR, other glucose transporters are regulated by the synergistic interactions between P4 and IFNt, with expression in the uterus being induced by P4 and further enhanced in response to conceptus secreted IFNt (Gao *et al.* 2009).

In addition to glucose transport, changes in the expression of mRNAs encoding several amino acid transporters in the endometrium in conjunction with changes to the amino acid profile of histotrophic secretions, allantoic fluids and amniotic fluids in response to P4 supplementation in pregnancy have been investigated. Progesterone supplementation on days 1.5–9 (i.m., 25 mg) increased the expression of the mRNA encoding the cationic amino acid arginine transporter *SLC7A2* in endometria on day 12 of pregnancy (Satterfield *et al.* 2010). A concurrent increase in the abundance of arginine in uterine flushings was observed on both days 9 and 12 of pregnancy in response to P4 supplementation. Similarly, there was a greater abundance of several neutral amino acids in uterine flushings on day 9 and an increase in the abundance of lysine (a basic amino acid) in uterine flushings on day 12 in response to P4 treatment (Satterfield *et al.* 2010). These differ from the findings by Hoskins and coworkers, in which P4 supplementation on days 1.5–8 (i.m., 25 mg) did not affect the expression of mRNAs encoding the amino acid transporter *SLC7A2* in endometria on days 9 and 12 of gestation but did increase the abundance of serine and isoleucine in uterine flushings on days 9 and 12, respectively (Hoskins *et al.* 2021). Halloran and coworkers reported that P4 supplementation on days 1.5–8 (i.m., 25 mg) increased the expression of mRNAs encoding cationic amino acid transporters (*SLC7A2* and *SLC7A1*) in the uterine endometrium on day 125 of pregnancy (Halloran *et al.* 2021). This was accompanied by an increase in the abundance of arginine in amniotic fluids, an increase in the abundance of aspartate in allantoic fluids and a decrease in the abundance of glutamate in allantoic fluids (Halloran *et al.* 2021). Arginine serves as a precursor for polyamines and nitric oxide (Wu 2009, Wu *et al.* 2013), which are important for conceptus and placental development (Halloran *et al.* 2019). P4 supplementation on days 1.5–8 (i.m., 25 mg) increased the expression of ornithine decarboxylase 1 (*ODC1*) and agmatinase (*AGMAT*) mRNAs, which encode enzymes that convert ornithine and agmatine, respectively, into polyamines in the uterine endometrium on day 125 of pregnancy (Halloran *et al.* 2021). Collectively, these data suggest that early P4 treatment may have a long-term programming effect on the endometrium that allows for alterations in the regulation of both amino acid transport and polyamine synthesis in late gestation (Halloran *et al.* 2021). As both amino acids and polyamines are critical for conceptus development (Wu 2009, Wu *et al.* 2013), these findings highlight the potential of exogenous P4 supplementation for enhancement of the availability of these important nutrients for utilization by the conceptus.

It has also been reported that exogenous P4 supplementation in early pregnancy can affect the expression of mRNAs encoding glucose and amino acid transporters in placentomes on day 125 of gestation. P4 supplementation increased the expression of *SLC7A1*, *SLC2A5* and *SLC2A8* mRNAs in placentomes, while the expression of *SLC2A1* and *SLC2A3* mRNAs was decreased in placentomes of P4-treated ewes compared to controls on day 125 of gestation (Halloran *et al.* 2021). The findings that supplementation of P4 in early pregnancy has effects on both endometrial and placental gene expression, as well as the abundance of nutrients in allantoic and amniotic fluids in late pregnancy, is an important concept that warrants further mechanistic investigation.

Tight and adherens junctions in the uterine endometrium facilitate cell–cell contact and adhesion in addition to regulating the passage of molecules through the paracellular space (Gonzalez-Mariscal *et al.* 2003). Supplementation with P4 on days 1.5–9 (i.m., 25 mg) decreased the abundance of both adherens and tight junction-associated proteins on days 9 and 12 of gestation compared to control ewes (Satterfield *et al.* 2007). It is hypothesized that this loss of tight junction integrity may lead to increased permeability of the uterine LE and sGE that allow for greater passage of nutrients and stromal-derived factors into the uterine lumen for use by the early conceptus (Satterfield *et al.* 2007). Further investigation is required to understand the potential impact of P4 supplementation on tight junction integrity and nutrient utero-placental transport.

During the peri-implantation period in sheep, both calcium and phosphate levels increase in uterine flushings (Gao *et al.* 2009). Furthermore, phosphate and calcium are more abundant in uterine flushings from pregnant animals compared to cyclic ewes during the mid-luteal phase of the estrous cycle (Choi *et al.* 2019). In addition, these changes in the mineral composition of uterine secretions are associated with alterations in the endometrial expression of molecules that regulate phosphate, calcium and vitamin D (Stenhouse *et al.* 2020, 2021*a*). This increase in mineral abundance in uterine flushings during the peri-implantation period is accompanied by alterations in the expression of several regulatory molecules for both calcium and phosphate (Stenhouse *et al.* 2020, 2021*a*,*b*). Considering the evidence that P4 may have an impact on nutrient transport and availability, it was hypothesized that P4 may also impact the regulation of vitamins and minerals during pregnancy. In fact, expression of several mRNAs involved in calcium, phosphate and vitamin D signaling was affected by P4 treatment (days 1.5–8, i.m., 25 mg) in the endometrium (days 12 and 125) and placentomes (day 125) (Stenhouse *et al.* 2021*a*). In the endometrium of ewes treated with P4 and IFNt to simulate pregnancy, a decrease in mRNA expression of fibroblast growth factor 23 (*FGF23*) and calcium-binding protein G (*S100G*) was detected when compared to control ewes given a P4 antagonist. Furthermore, it has been suggested that tissue nonspecific alkaline phosphatase (TNSALP; encoded by the *ALPL* gene), an important regulator of utero-placental phosphate availability in sheep, is altered by P4 treatment (Stenhouse *et al.* 2024). P4 treatment on days 1.5–8 (i.m., 25 mg) resulted in increased expression of *ALPL* mRNA in the endometrium on day 12 of gestation (Stenhouse *et al.* 2024). Interestingly, expression of *ALPL* mRNA was significantly decreased in the endometrium of P4-treated singleton pregnancies on day 125 of gestation compared to P4-treated twin pregnancies, suggesting an interaction between litter size and treatment (Stenhouse *et al.* 2024). TNSALP enzymatic activity also appears to be increased in the epithelia, stratum compactum stroma and endothelial cells in the endometrium of P4-treated ewes on day 12 (Stenhouse *et al.* 2024). In addition, P4 treatment appeared to increase TNSALP enzymatic activity in the endothelium and caruncular stromal tissue in placentomes on day 125 (Stenhouse *et al.* 2024). Collectively, these data suggest that P4 may have a regulatory role in mineral homeostasis during pregnancy, although this area warrants further investigation.

Several studies have reported effects of exogenous P4 supplementation on pre-implantation conceptus development; however, to date, there are limited reports on the impact of exogenous P4 on fetal growth and development. Studies investigating the impact of early progesterone supplementation (days 1–3, CIDR 300 mg) on conceptus development in mid-gestation (day 76) reported increased fetal weight, crown–rump length and weights of fetal organs, including the brain, thyroid, heart, lungs, liver, spleen and kidney (Kleemann *et al.* 1994, 2001). Fetal organ weight increased proportionally to the total fetal weight increase, except the heart and brain, which had disproportionately large weight increases in fetuses from P4-treated pregnancies (Kleemann *et al.* 2001). Progesterone supplementation did not affect placental weight. However, the volume of the chorionic membrane and maternal crypts increased (Kleemann *et al.* 2001). These findings are complemented by work from Wallace and coworkers, in which low-dose P4 treatment (i.m., 12.5 mg) on days 5–55 of gestation increased birth weight in adolescent ewes with a high nutritional intake (Wallace *et al.* 2003). It has been suggested that overfed adolescent ewes tend to divert nutrients to self-growth at the cost of the developing fetus, leading to low birth weights (Wallace *et al.* 1996). Low endogenous P4 levels are also characteristic of overnourished pregnant ewes (Wallace *et al.* 1994). It is possible that exogenous P4 administration may mitigate these issues. In contrast, supplementation with P4 (i.m., 25 mg) on days 1.5–8 did not impact fetal or placental growth on day 125 of pregnancy (Halloran *et al.* 2021). It is important to note that while these studies appear to be contradictory, the methodologies of the experiments are quite varied. It is possible that dosage, timing and mode of administration influence final outcomes. These studies also investigated different end points ranging from mid- to late gestation. The fetal sheep experiences exponential growth period starting at mid-gestation and continuing through late pregnancy (Bazer *et al.* 2012). In addition, placental growth is known to precede fetal growth under normal physiological conditions (Bazer *et al.* 2012). Further studies are required to determine the impact of P4 supplementation on fetal growth in mid- and late gestation and how this impacts postnatal animal health.

Although P4 supplementation appears to be a promising approach to enhancing reproductive performance in sheep, further research is warranted for assessing the impact of timing, dosage and method of supplementation. There may also be a change in effect of P4 supplementation based on fetal sex and litter size. Several of the explored mRNAs change the amount of expression based on singleton vs twin pregnancies and fetal sex (Halloran *et al.* 2021). In addition, there is evidence to suggest that the magnitude of the effect of P4 may be different depending on pregnancy type and fetal sex (Halloran *et al.* 2021, Stenhouse *et al.* 2024). These issues need further exploration to optimize P4 supplementation as a strategy for improved reproductive performance in sheep.

### Pigs

Circulatory P4 levels during the first week of gestation are positively associated with embryo survival in pigs (Pharazyn *et al.* 1991, Jindal *et al.* 1997). Thus, it could be speculated that supplementation of P4 in early pregnancy may enhance embryonic/fetal survival. However, reports of the impact of exogenous P4 supplementation on pregnancy outcomes and conceptus development in pigs have yielded highly variable results, with studies suggesting that P4 supplementation has no effects, negative effects or positive effects on pregnancy outcomes and conceptus development. Findings of some of the essential studies investigating the impact of exogenous P4 supplementation on pregnancy outcomes and conceptus development are summarized in Table 1.

**Table 1:** Studies investigating the impact of exogenous progesterone on pregnancy outcomes and conceptus development in pigs.

Study	Route of administration	Period of supplementation and frequency	Dosage	End point investigated	Effects
Ashworth (1991)	i.m. injections	Days 4–30, daily	50 mg	Day 30	More fetuses in *ad libitum*-fed gilts supplemented with progesterone; greater allantoic fluid volume and total glucose abundance in allantoic fluid
Jindal *et al.* 1997	i.m. injections	24, 36, 48, 60, 72 and 84 h after the onset of standing estrus	75 mg	Day 28	Greater embryonic survival
Mao & Foxcroft (1998)	i.m. injections	Every 12 h for 36–96 h after the onset of standing estrus	2 mg of P4/kg	Day 28	Decreased litter size and percentage embryonic survival
Muro *et al.* (2020)	i.m. injection*	Day 6	2.15 mg/kg body weight	Day 28	No effect of injections or oral supplementation on pregnancy rate, embryo survival or endometrial vascular density
	Oral^†^	Days 6–12, daily	20 mg		Gilts – supplementation (both injection and oral) resulted in smaller embryos. Greater mean and total glandular areas in supplemented (both injection and oral) endometria
					Sows – oral supplementation had larger embryos. Decreased total glandular area in oral supplemented endometria
Muro *et al.* (2022)	Oral^†^	Days 6–12, daily	20 mg	Farrowing	Increased total number of piglets born, increased number of mummified fetuses, increased number of piglets born alive (and decreased stillbirth rate), increased placental weight and decreased number of piglets born weighing <800 g
Soede *et al.* (2012)	Oral^†^	Days 1–4 or 2–4, daily	20 mg	Day 42	Decreased pregnancy rate and litter size. Initiation of supplementation close to the timing of ovulation augmented the negative effects of progesterone supplementation when compared to gilts that initiated supplementation approx. 12 h later
		Days 4 and 6	10 mg or 20 mg	Farrowing	20 mg tended to have a smaller litter size
Spies *et al.* (1959)	s.c. injections	Days 0–4, 0–18, 0–25, 4–18, 4–25 or 18–25, daily	1 mg/lb body weight	Day 18 or 25	Progesterone supplementation from day of mating had detrimental effects on embryonic survival. Progesterone supplementation from day 4 or 18 did not have a deleterious effect on embryonic survival. Progesterone supplementation decreased corpus luteal weight
Szymanska & Blitek (2016)	i.m. injections	Days 3–10, daily	25 mg/100 kg body weight on days 3 and 4; 50 mg/100 kg body weight on days 5–10	Day 12	Increased uterine weight. No alterations in conceptus morphology. Greater total protein abundance and 6-keto PGF1α in uterine flushings. Greater expression of prostaglandin-endoperoxide synthase 2, microsomal PGE2 synthase and vascular endothelial growth factor A mRNA in the endometrium
Vallet *et al.* (1998)	i.m. injections	Days 2 and 3	200 mg	Days 10, 11, 12, 13 and 15	Increase in total protein in uterine flushings, increased intrauterine retinol binding protein abundance and elevated estradiol abundance in flushings on day 11
Vallet & Christenson (2004)	i.m. injections	Days 2 and 3	200 mg	Day 105	Decreased litter size
		Days 2 and 3	200 mg	Farrowing	Decreased pregnancy rates. No effect on number of live or still born piglets in gilts that maintained pregnancies. Decreased gestation length
Yu *et al.* (1997)	i.m. injections	Days 2–14, daily; days 4–14, daily	50 mg	Days 25–28	No effect on pregnancy rate, embryo number and survival
Yu *et al.* (1999)	i.m. injections	Days 2–4, daily; days 4–14, daily	50 mg	Days 25–28	No effect on embryo survival or endometrial IGF1 mRNA expression

i.m, intramuscular; s.c., subcutaneous

* Long-acting progesterone.

^†^ Progesterone analog (altrenogest).

As described for sheep, timing and dosage of P4 supplementation are important to consider when interpreting these findings. Supplementation of P4 (i.m., 2 mg of P4/kg) every 12 h for 36–96 h after the onset of standing estrus decreased litter size and the percentage of embryonic survival on day 28 (Mao & Foxcroft 1998). Similarly, supplementation of P4 from the day of mating (subcutaneous injection, 1 mg/lb body weight) until days 4, 18 or 25 decreased embryonic survival on days 18 and 25 (Spies *et al.* 1959). In contrast, Jindal and coworkers demonstrated that supplementation of P4 (i.m., 75 mg) at 24, 36, 48, 60, 72 and 84 h after the onset of standing estrus increased embryonic survival on day 28 (Jindal *et al.* 1997).

It could be speculated that initiating P4 supplementation too close to the onset of estrus, which is characterized as a highly estrogenic state, may have adverse effects on conceptus development and survival. Thus, it could be speculated that if the initiation of P4 supplementation is postponed, this could be more beneficial for conceptus development. However, it has also been reported that gilts supplemented with P4 (200 mg/day) on days 2 and 3 of gestation had decreased pregnancy rates compared to control gilts (Vallet & Christenson 2004). No impact of P4 supplementation on the number of live or stillborn piglets or the incidence of small or large weight piglets at farrowing was reported in the gilts that established and maintained pregnancies, although gestation length was significantly shorter.

Supplementation of P4 on days 3–10 of gestation did not alter conceptus morphology on day 12 of gestation, with conceptuses of filamentous morphology present in both control and supplemented gilts (Szymanska & Blitek 2016), suggesting that there was no impact on conceptus development at this stage of gestation. Similarly, supplementation (i.m., 50 mg daily) from either 2 or 4 days after estrus detection until day 14 had no impact on fetal survival on days 25–28 of gestation (Yu *et al.* 1997, 1999). In contrast, supplementation of P4 to gilts on days 4–30 of gestation (i.m., 50 mg/day) increased the number of viable fetuses on day 30 of gestation (Ashworth 1991). It could be speculated that expanding the window of P4 supplementation to include the peri-implantation period and initiation of placentation (Marrable 1971, Johnson *et al.* 2021) has beneficial effects on implantation and placentation.

As described above, P4 is an important regulator of uterine secretions, and thus, it could be speculated that exogenous P4 supplementation would alter the secretory capacity of the porcine uterus. Gilts that received daily injections of P4 on days 3–10 of gestation had a greater total protein content in the uterine lumen on day 12 of gestation than control gilts (Szymanska & Blitek 2016), suggesting enhanced uterine secretions and thus greater nutrient availability for the rapidly proliferating and elongating conceptuses. Similarly, P4 supplementation of gilts (i.m., 200 mg/day) on days 2 and 3 after breeding resulted in greater total protein abundance in uterine flushings on days 10, 11, 12, 13 and 15 of gestation (Vallet *et al.* 1998). However, P4 supplementation (i.m., 200 mg) of gilts that were ovariectomized on day 12 for 28 days did not result in hyperplasia or hypertrophy of the uterine GE but did increase the height of the uterine LE (Bailey *et al.* 2010). This could indicate that perhaps the observed effects of P4 supplementation on uterine flushing composition occur due to alterations in LE secretory activity.

In addition to the importance of histotrophic secretions from the uterine epithelia for conceptus development, there is a well-established link between utero-placental vascularity and conceptus development in livestock species (Reynolds & Redmer 1995, 2001, Reynolds *et al.* 2005, Stenhouse *et al.* 2019*a*,*b*). Several studies suggest that P4 supplementation may have a positive effect on angiogenesis in porcine utero-placental tissues. Gilts that received daily injections of P4 on days 3–10 of gestation had greater endometrial expression of vascular endothelial growth factor (*VEGF*) mRNA on day 12 of gestation (Szymanska & Blitek 2016). Similarly, ovariectomized gilts treated with P4 (0.83 mg/kg body weight daily, days 16–19) had greater endometrial expression of *VEGF* mRNA than control gilts (Welter *et al.* 2003). Likewise, supplementation of gilts ovariectomized on day 12 with P4 (i.m., 200 mg) for 28 days resulted in an increase in vascularity in the deep uterine endometrium (Bailey *et al.* 2010). However, this was not observed in the shallow endometrium. Collectively, these findings suggest that supplementation of pigs with P4 has the potential to increase angiogenesis and vascularity of the uterus, thereby potentially increasing nutrient transport.

As described earlier, glucose is an important energy source for the rapidly proliferating conceptus. Supplementation of P4 to gilts on days 4–30 of gestation (i.m., 50 mg/day) increased both the allantoic fluid volume and the total abundance of glucose in allantoic fluid on day 30 of gestation (Ashworth 1991). Similarly, P4 supplementation of ovariectomized gilts from day 12 of the estrous cycle through daily injections (i.m., 200 mg) for 28 days resulted in increased endometrial expression of *SLC2A1* mRNA, which was localized to the uterine LE (Kramer *et al.* 2020). These findings suggest that P4 supplementation may be a strategy to enhance glucose transport in gilts, which may be a useful strategy to enhance conceptus development.

Appropriate secretion of the maternal recognition of pregnancy signal by conceptuses is crucial for the establishment of pregnancy. Gilts supplemented with P4 (i.m., 200 mg on days 2 and 3 after estrus) had more estradiol in uterine flushings on day 11 (Vallet *et al.* 1998). This study did not report whether the P4 treatment had any impact on conceptus morphology, but it could be speculated that there may be a significantly greater number of estradiol secreting cells in the conceptus or increased production of estradiol per cell in response to the P4 treatment, which could have significant impacts on maternal recognition of pregnancy, the regulation of endometrial function and conceptus development (Geisert *et al.* 2024).

Progesterone supplementation of ovariectomized gilts from day 12 of the estrous cycle received daily injections of P4 (i.m., 200 mg) for 28 days resulted in increased uterine weight and length, accompanied by a thicker endometrium and myometrium on day 40 (Bailey *et al.* 2010). Progesterone did not induce uterine gland hyperplasia or hypertrophy, suggesting that P4 alone is not sufficient to induce the changes in these cell types associated with pregnancy progression. Adhesion and tight junction complexes are essential for the establishment and maintenance of pregnancy in livestock species. Progesterone supplementation resulted in the absence of Dolichos biflorus agglutinin lectin staining in the LE and low levels of expression in the GE. Similarly, decreased expression of the αv integrin subunit was observed at the apical surface of uterine epithelia in P4-treated gilts compared to controls. Bailey *et al.* investigated the potential impact of P4 supplementation on the integrity of tight junction complexes. The organization of zonula occludens-1 and 2 (ZO-1 and ZO-2) remained unchanged between control and P4-treated gilts. Progesterone did not affect the distribution or intensity of claudin-3 immunostaining. In contrast, claudin-4 protein was only found in the uteri of P4-treated gilts, localizing to basal and lateral surfaces of both uterine epithelia. Similarly, P4 supplementation induced the expression of the α2β1 integrin heterodimer along the basal and lateral surfaces of LE and GE, which was absent in controls. Notably, P4 supplementation resulted in vimentin protein expression at the basal and lower third of the lateral surfaces of the uterine LE. These findings suggest that exogenous P4 supplementation may induce significant changes in the structure of the uterine extracellular matrix, which could have significant consequences for adhesion and subsequent function of the utero-placental interface.

While many studies investigating exogenous P4 supplementation in livestock species have utilized multiple i.m. injections, this strategy would be labor-intensive for on-farm application. Therefore, there is a critical need to evaluate alternative less labor-intensive approaches to provide P4 to sows. One such strategy is the utilization of a single long-lasting progesterone injection. Muro and coworkers supplemented gilts and sows with a single injection (2.15 mg/kg body weight, i.m.) of a long-lasting progesterone on day 6 of pregnancy and assessed conceptus development on day 28 (Muro *et al.* 2020). This single injection had no effect on pregnancy rate, embryo survival or endometrial vascular density on day 28 of gestation. However, gilts supplemented with long-lasting P4 on day 6 had smaller embryos accompanied by a larger area of the endometrium composed of uterine glands on day 28.

An alternative strategy that can be utilized for P4 supplementation in swine is oral supplementation with altrenogest, a P4 analog. While this has proved an effective tool for the synchronization of estrous cyclicity, there are conflicting reports regarding the potential of altrenogest supplementation during gestation. Progesterone supplementation (20 mg/d) during the first four days of gestation resulted in a decrease in both pregnancy rate and litter size on day 42 of gestation when compared with control gilts (Soede *et al.* 2012). Interestingly, ultrasound examination was utilized in this study every 12 h to determine the timing of ovulation, which allowed demonstration that the initiation of supplementation close to the timing of ovulation further enhanced the negative effects of P4 supplementation when compared to gilts that initiated supplementation approximately 12 h later. While the exact mechanism of this difference in response is not fully understood, it could be speculated that altrenogest treatment may alter uterine contractions, which could alter spermatozoa transport through the female reproductive tract, thereby reducing the number of spermatozoa present in the oviduct leading to a decrease in fertilization. In addition, it has been suggested that low accessory sperm count (the number of sperm cells in the zona pellucida) is associated with a greater embryonic diversity, which is known to be associated with enhanced mortality during the peri-implantation period (Pope *et al.* 1990, Soede & Kemp 1993, Kemp & Soede 1997).

Altrenogest supplementation (20 mg/d) on days 6–12 did not affect pregnancy rate, embryonic survival or endometrial vascular density on day 28 of gestation (Muro *et al.* 2020). However, supplementation of gilts resulted in smaller embryos and a larger area of the endometrium composed of uterine glands on day 28. In contrast, sows had larger embryos and a decreased endometrial glandular area on day 28. As no effect on pregnancy rate was observed, this could indicate that oral supplementation with altrenogest on days 6–12 may have beneficial effects on embryo development in sows but not gilts. However, further studies are needed to ascertain whether this effect would be observed in late gestation or at farrowing. In addition, the impact on uterine glandular area is an important consideration as this would be speculated to influence histotrophic secretions and thus nutrient availability for conceptus utilization.

To fully assess the impact of this supplementation strategy for on-farm utilization, it is important to assess the impact of the supplementation on litter characteristics at farrowing. Supplementation of gilts with 20 mg/day of altrenogest on days 4 and 6 after estrus onset resulted in a tendency for a smaller litter size at farrowing (Soede *et al.* 2012). In contrast, altrenogest supplementation (20 mg/day) of sows (parity 1–8) on days 6–12 increased the total number of piglets born, increased the number of piglets born alive, decreased the incidence of stillbirths, decreased the number of piglets born weighing <800 g and increased the placental weight (Muro *et al.* 2022).

Genetic selection (Knap *et al.* 2023) has successfully increased litter size by 59% over the past 50 years in the US (https://www.nass.usda.gov/, accessed 03/04/24), a trend that has been observed worldwide. As such, it could be speculated that the endocrinology of the modern hyperprolific sow and their response to exogenous P4 may be different to the sows used in some of the original papers investigating this topic. This may be a further factor to consider when investigating the potential of exogenous P4 supplementation on pregnancy outcomes and conceptus development and is an aspect that warrants further investigation.

## Conclusions

**Figure 1:**
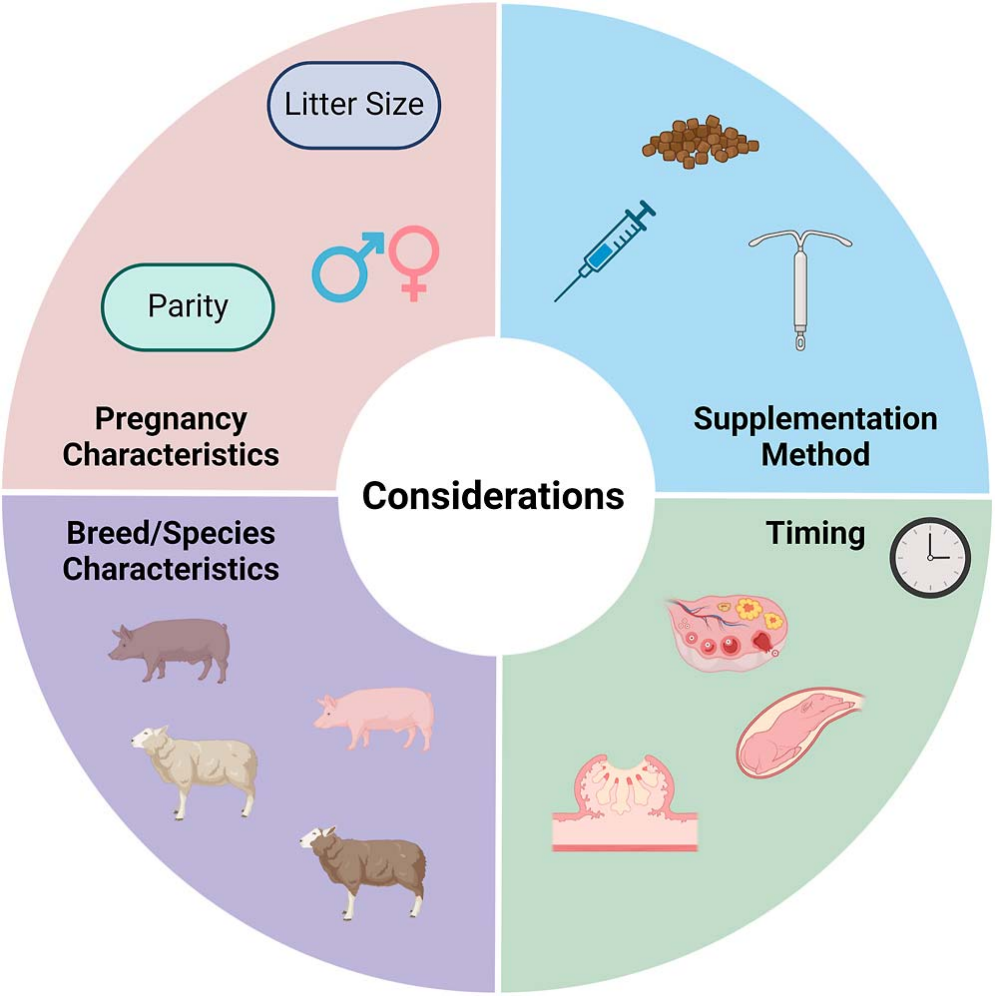
Considerations for progesterone supplementation trials in livestock species. Studies to date have shown substantially varied outcomes of progesterone supplementation, and this can be due to many factors. Examples of common considerations for designing and interpreting studies include i) supplementation method – injection (intramuscular, subcutaneous or intravenous), in feed or by hormone-releasing devices; ii) timing – stage of the ovarian cycle (during the period(s) of rapid placental or fetal development); iii) breed/species characteristics – different species or breeds may respond differently to supplementation; and iv) pregnancy characteristics, including litter size, fetal sex, and parity of the dam. Figure made with BioRender.

Progesterone is an essential hormone for the establishment and maintenance of pregnancy, which has potential to be supplemented as a strategy to enhance reproductive performance in livestock species. Studies undertaken to determine the impact of exogenous P4 supplementation on pregnancy outcomes and conceptus development in sheep and pigs have yielded highly variable results, with studies suggesting that P4 supplementation has no effect on, has a negative effect on or enhances conceptus development. The variation in findings may be attributed to multiple factors, including timing, dosage, method of supplementation, breed-/species-specific characteristics, and characteristics of that pregnancy such as litter size, fetal sex and parity of the mother (Fig. 1). In addition, genetic selection has been performed to enhance reproductive efficiency in both species over recent decades, making it difficult to translate the findings from earlier publications in this field to modern-day breeds. Further studies are needed to fully understand the impact of exogenous P4 supplementation on pregnancy outcomes and conceptus development and on lactation, offspring health and reproductive performance postnatally.

## Declaration of interest

The authors declare that there is no conflict of interest that could be perceived as prejudicing the impartiality of the research reported. CS is an Associate Editor for *Reproduction & Fertility* and was not involved in the review or editorial process associated with this paper.

## Funding

This work was supported by the USDA National Institute of Food and Agriculturehttps://doi.org/10.13039/100005825 and Multistate/Regional Research Appropriations under Project PEN04775 and Accession number 7001112, the USDA National Institute of Food and Agriculture and Hatch Appropriations under Project PEN04995 and Accession number 7007748 and NIHhttps://doi.org/10.13039/100000002 Grant T32GM154124.

## Author contribution statement

All authors edited, reviewed and approved the final manuscript.
